# Quantitative Sensory Testing Predicts Pregabalin Efficacy in Painful Chronic Pancreatitis

**DOI:** 10.1371/journal.pone.0057963

**Published:** 2013-03-01

**Authors:** Søren S. Olesen, Carina Graversen, Stefan A. W. Bouwense, Harry van Goor, Oliver H. G. Wilder-Smith, Asbjørn M. Drewes

**Affiliations:** 1 Mech-Sense, Department of Gastroenterology, Aalborg Hospital, Aarhus University Hospital, Aalborg, Denmark; 2 Mech-Sense, Department of Radiology, Aalborg Hospital, Aarhus University Hospital, Aalborg, Denmark; 3 Department of Neurorehabilitation Engineering, Bernstein Center for Computational Neuroscience, University Medical Center Göttingen, Georg-August University, Göttingen, Germany; 4 Pain and Nociception Neuroscience Research Group, Department of Surgery, Radboud University Nijmegen Medical Centre, Nijmegen, The Netherlands; 5 Pain and Nociception Neuroscience Research Group, Department of Anaesthesiology, Pain and Palliative Care, Radboud University Nijmegen Medical Centre, Nijmegen, The Netherlands; 6 Center for Sensory-Motor Interaction (SMI), Department of Health Science and Technology, Aalborg University, Aalborg, Denmark; University of California, United States of America

## Abstract

**Background:**

A major problem in pain medicine is the lack of knowledge about which treatment suits a specific patient. We tested the ability of quantitative sensory testing to predict the analgesic effect of pregabalin and placebo in patients with chronic pancreatitis.

**Methods:**

Sixty-four patients with painful chronic pancreatitis received pregabalin (150–300 mg BID) or matching placebo for three consecutive weeks. Analgesic effect was documented in a pain diary based on a visual analogue scale. Responders were defined as patients with a reduction in clinical pain score of 30% or more after three weeks of study treatment compared to baseline recordings. Prior to study medication, pain thresholds to electric skin and pressure stimulation were measured in dermatomes T10 (pancreatic area) and C5 (control area). To eliminate inter-subject differences in absolute pain thresholds an index of sensitivity between stimulation areas was determined (ratio of pain detection thresholds in pancreatic versus control area, ePDT ratio). Pain modulation was recorded by a conditioned pain modulation paradigm. A support vector machine was used to screen sensory parameters for their predictive power of pregabalin efficacy.

**Results:**

The pregabalin responders group was hypersensitive to electric tetanic stimulation of the pancreatic area (ePDT ratio 1.2 (0.9–1.3)) compared to non-responders group (ePDT ratio: 1.6 (1.5–2.0)) (*P* = 0.001). The electrical pain detection ratio was predictive for pregabalin effect with a classification accuracy of 83.9% (*P* = 0.007). The corresponding sensitivity was 87.5% and specificity was 80.0%. No other parameters were predictive of pregabalin or placebo efficacy.

**Conclusions:**

The present study provides first evidence that quantitative sensory testing predicts the analgesic effect of pregabalin in patients with painful chronic pancreatitis. The method can be used to tailor pain medication based on patient’s individual sensory profile and thus comprises a significant step towards personalized pain medicine.

## Introduction

A major problem for pain treatment is the lack of knowledge about which treatment best suits a specific patient. This is particularly important in patients with chronic pancreatitis (CP) due to complex and multifactorial pain aetiology, involving many factors including structural abnormalities of the pancreatic gland as well as abnormalities of peripheral and central pain processing [1]. Chronic pain in CP is also associated with malnutrition, narcotic addiction, physical and emotional disability and major socioeconomic problems, which further complicates and blurs the clinical evaluation of treatment outcome [2], [3]. For these reasons pain management in CP is challenging and often leads to a time-consuming and unsatisfactory approach to treatment with an unpredictable outcome.

The complex pain aetiology and wide variability in group treatment outcome makes it important to identify biomarkers linked to outcomes of pain treatment in individual patients. Quantitative sensory testing (QST) has in some studies been able to predict treatment outcome of analgesic therapy in chronic pain disorders [4]–[6]. It provides information on sensory function at the peripheral and central level of the nervous system by recording the subjects’ responses to different external stimuli of controlled intensity [7]. In painful CP, changes in pain processing affect second order neurones in the central nervous system (CNS) receiving convergent visceral and somatic afferent information. Hence, sensory information from *static* QST of skin dermatomes in the upper abdominal area can indirectly be used to obtain information about CNS neuroplasticity following increased barrage from pancreatic sensory afferents [8], [9]. In addition, QST can be used to gain information on the *dynamic* function of the sensory system, including descending inhibitory and facilitatory influences from the brain stem and higher cortical levels, e.g. by using conditioned pain modulation (CPM) paradigms [10].

A recent randomised, placebo controlled, clinical trial of pregabalin in patients with painful CP provided the opportunity to test a putative link between pre-treatment QST measurements and effectiveness of pregabalin and placebo in treating the pain of CP [11]. Pregabalin has effectively been used to treat various chronic pain disorders, including diabetic neuropathy [12], post herpetic neuralgia [13], and neuropathic pain of central origin [14]. It binds selectively to voltage dependent calcium channels and blocks influx of calcium into presynaptic nerve terminals [15]. This reduces release of excitatory neurotransmitters on spinal neurons, and in turn reduces neuronal excitability and upstream transmission in the central nervous system [16].

We hypothesized pregabalin to be more efficacious in CP patients characterized by central sensitization, as expressed by increased responsiveness to static QST in the upper abdominal region. In turn, we expected that descending pain modulation (i.e. dynamic QST) would not be associated with pregabalin efficacy, as pregabalin do not exert its primary effect through descending pain modulation [17]. The aim of this study was to test this hypothesis by testing the ability of static and dynamic QST to predict pregabalin and placebo efficacy in patients with painful CP.

## Methods

### Study Patients

Patients were recruited from an investigator initiated double-blinded, placebo-controlled, parallel-group study of pregabalin for painful CP conducted in Denmark (Department of Gastroenterology and Hepatology, Aalborg Hospital, Aarhus University Hospital) and the Netherlands (Department of Surgery, Radboud University Nijmegen Medical Center) [11]. The present study investigates the link between baseline QST measurements and analgesic efficacy of pregabalin or placebo.

Key inclusion criteria were a diagnosis of CP based on The Mayo Clinic Diagnostic Criteria and chronic abdominal pain typical for pancreatitis, meeting the criteria for chronic pain (pain ≥3 days per week in at least 3 months) and by the patient considered severe enough for medical treatment [18]. Patients taking concomitant analgesic medication and expected to stay on a stable regime during the trial were allowed to enter the study. Patients were excluded from the study if they had a painful condition other than CP or were previously operated in the areas subjected to QST. Testing in females was not standardized with regard to phase of the menstrual cycle because all pancreatitis patients had amenorrhea or were postmenopausal.

The responsible Ethical Committees in both countries approved the study and all patients provided written informed consent prior to investigation.

### Study Design and Treatment

The study consisted of a 3-week period of pregabalin or placebo treatment titrated to analgesic effect and tolerability. In each patient, a QST session was performed before starting study medication. The session consisted of static (pain thresholds to pressure and electric tetanic stimulation) and dynamic QST (conditioned pain modulation, CPM). A detailed patient history was obtained to determine pain localization, intensity of ongoing pain (assessed by a 0–10 visual analogue scale (VAS)), and use of analgesics. Patients were then instructed and trained in VAS ratings to determine pain thresholds and the CPM paradigm. A 30 minute pause allowed the pain system to re-establish baseline conditions and was followed by the QST test session.

After QST testing patients received escalating doses of pregabalin (300 to 600 mg/day) or matching placebo capsules for three weeks. Daily dosages were split into two equivalent doses, one administered in the morning and one in the evening. If unacceptable side effects were experienced by the patient, a single downward dose titration was allowed, after which the patient remained on the final dosage during the remainder of the study period. A minimum end dose of 300 mg/day was required; otherwise the subject was withdrawn from the study.

### Clinical Outcome

The clinical endpoint was the percentage change in daily *average* pain intensity after three weeks of treatment compared to baseline. The average daily pain scores (i.e. the *average* pain score for the last 24 hours) were collected by a pain diary based on a visual analogue scale (VAS), where 0 = no pain and 10 = worst pain imaginable. The baseline pain score was calculated as the average daily pain score during the week prior to randomization (i.e. no study medication). The response to study medication was calculated as the average daily pain score during the last week of treatment. Responders to treatment (pregabalin or placebo) were defined as patients with a reduction in clinical pain score of 30% or more after three weeks of treatment compared to baseline recordings.

### Quantitative Sensory Testing

#### Pressure and electric tetanic threshold testing

Threshold testing took place using a standard temporal test sequence, which has been described in detail previously [9], [19]. *Pressure* pain thresholds were obtained for muscles overlying bone by pressing a handheld electronic pressure algometer (Somedic AB, Stockholm, Sweden). The probe had a surface area of 1 cm^2^ and the pressure was increased at a rate of 30 kPa/sec. The lower neck (C_5_ dermatome) and the upper abdominal area (ventral Th_10_ dermatome) on the dominant body side were stimulated. The upper abdominal area was chosen because dorsal horn neurons receiving painful stimuli from this skin area also receive nociceptive stimuli from the pancreas (i.e. pancreatic area). In contrast, the lower neck area was chosen because the nociceptive pathways from this area are separated from those coming from the pancreas at both peripheral and spinal levels (i.e. control area). For each stimulation site, pain detection thresholds were measured (pPDT, stimulation just becomes painful).

Pain thresholds to *electric* constant current skin stimulation (ePDT) (Digistim; Biometer A/S, Copenhagen, Denmark; tetanic stimulation at 100 Hz, 0.2 ms square waves, self-adhesive electrodes 3 cm apart) were measured on the same sites as for pressure stimulation.

To obtain an index of sensitivity between stimulation areas (i.e. pancreatic area vs. control area) the relation between thresholds was determined as pPDT ratio (pPDT pancreas/pPDT control) and ePDT ratio (ePDT pancreas/ePDT control). This eliminated inter-subject differences in absolute pain thresholds and thereby provided a measure of “pancreatic sensitivity”.

#### Conditioned pain modulation (CPM)

The CPM paradigm was performed to test the ability of the patient to generate descending inhibitory modulation. CPM is a clinically measurable form of descending pain modulation, which can be induced experimentally by a conditioning stimulus (e.g. the cold pressor test) and quantified by applying a test-stimulation before and after its induction [10].

*Conditioning stimulus - the cold pressor task*: The right hand was immersed in cooled water (2.0°C±0.3°C, continuously stirred by a pump). The patients were told to remove the hand from the water after 3 minutes of immersion - or earlier if the pain was considered to be intolerable. The duration of cold pressor stimulation was measured for each patient (cold pressor).

*Test stimulus - somatic pressure stimulation*: Pain tolerance thresholds (pPTT, painfulness of stimulation just becomes intolerable) was determined on the quadriceps muscle 5 cm proximal to the patella (corresponding to the L4 dermatome) before the cold pressor task and immediately after its completion. The same pressure algometer as for the static QST paradigm was used. The *CPM effect* was defined as the relative change (%) in pPTT.

### Prediction Algorithm

**Figure 1:**
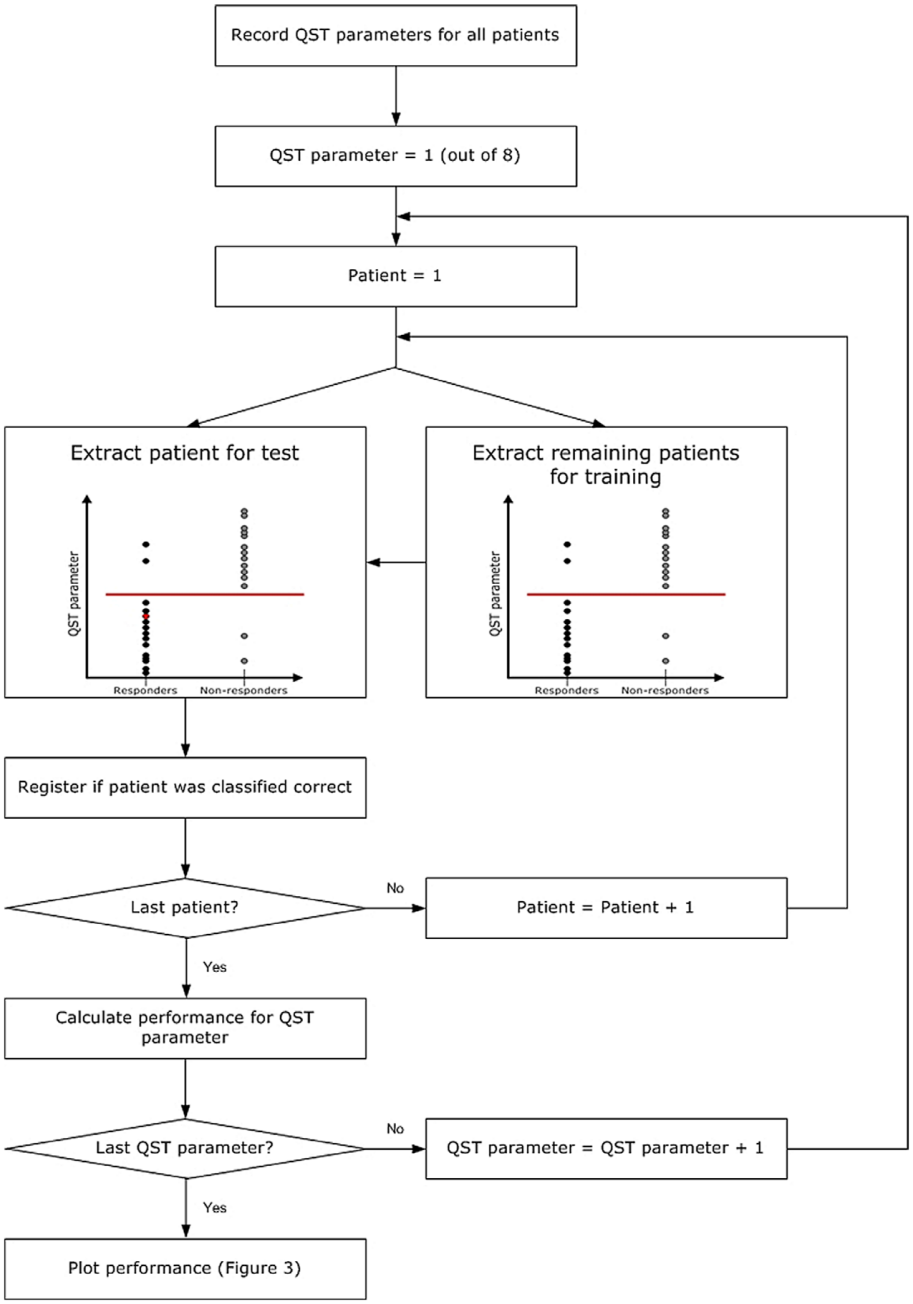
Schematic illustration of the screening procedure with the support vector machine (SVM). The basic principle of the SVM is to calculate a threshold which optimally discriminates responders and non-responders. The system performance is calculated by a leave-one-out strategy (LOU) for each of the quantitative sensory testing (QST) parameters. The LOU strategy is a cross-validation procedure where the system tests each patient individually by training the system on all other patients. Hence, the patient under test has not contributed to define the threshold. After testing each of the patients, the system performance is calculated as the number of correctly classified patient out of the total number of patients, which reflects the predictive capacity of the QST parameter.

The prediction algorithm was utilized by a support vector machine (SVM) based on machine learning [20]. SVM is a binary classifier, which separates data from two groups by an optimal separating threshold as illustrated in figure 1. The SVM was chosen since it has been used for prediction in other studies of biological data, and furthermore has the advantage that the objective threshold is calculated without any *a priori* assumptions of the discriminative features [21], [22].

The SVM was applied to discriminate responders and non-responders. As prediction studies should be based on analysis of individual patients rather than statistical analysis of differences between study groups, the various QST measurements should be tested independently. Hence, the SVM was first applied to screen the following parameters for discriminative capacity by a leave-one-out approach (figure 1): 1) pPDT pancreas, 2) pPDT control, 3) pPDT ratio, 4) ePDT pancreas, 5) ePDT control, 6) ePDT ratio, 7) cold pressor time, and 8) CPM effect.

After screening the parameters, the QST measurements leading to the maximum discrimination of the responders and non-responders were considered the optimal prediction model. The model was then assessed with respect to statistical significance to predict if a patient would respond to pregabalin or placebo treatment based on baseline QST assessment. Finally, the SVM was applied to calculate the optimal separation between patients.

### Statistical Analysis

All data are presented as medians with interquartile ranges unless otherwise indicated. Demographics, clinical data, and baseline QST measurements for responders and non-responders were compared by Wilcoxon signed rank test, Mann-Whitney *U* test and Fisher’s exact test as appropriate. The significance of the SVM discrimination between responders and non-responders was analyzed by a Fisher’s exact test. A *P*-value <0.05 was considered as an indication of statistical significance. In case of multiple comparisons the *P*-value was adjusted to <0.01 [23], [24]. The software package STATA version 11.2 (StataCorp LP, College Station, Texas, USA) was used for statistical calculations.

## Results

Sixty-four patients with painful CP were enrolled for the study (i.e. randomized to receive pregabalin or placebo in the original RCT [11]). Three patients in the pregabalin group and one patient in the placebo group had incomplete pain diary data at follow up and could not be classified as responders or non-responders. Consequently, these subjects were excluded from the further per-protocol analysis (n = 60). Clinical and demographic characteristics of patients with stratification of data for responders and non-responders are provided in Table 1. Groups were balanced with respect to clinical and demographic pre-treatment characteristics (all *P*>0.10).

**Table 1:** Baseline demographic and clinical characteristics.

		Pregabalin		Placebo	
		Responders (n = 16)	Non-responders (n = 15)	Responders (n = 12)	Non-responders (n = 17)
Age (years)		52 (50–59)	49 (43–57)	54 (41–63)	59 (49–64)
Males - no. (%)		9 (56)	8 (53)	9 (75)	10 (59)
Aetiology - no. (%)	Toxic (alcohol)	6 (38)	9 (60)	9 (75)	7 (41)
	Other	10 (63)	6 (40)	3 (25)	10 (59)
Diary pain score (VAS 0–10)		3.6 (2.6–5.1)	4.3 (2.4–6.5)	3.6 (2.1–5.1)	4.4 (2.1–5.0)
Concomitant analgesics – no. (%)†	None	2 (13)	1 (7)	2 (17)	0 (0)
	Weak analgesics	4 (25)	3 (20)	3 (25)	8 (47)
	Strong analgesics	10 (63)	11 (73)	7 (58)	9 (53)
Opioid equipotency (mg/day)		71 (4–127)	80 (10–180)	45 (23–135)	48 (8–120)
Duration of CP (months)		83 (54–131)	117 (100–166	151 (77–212	84 (73–112)
Diabetes mellitus - no. (%)		7 (44)	3 (20)	6 (50)	4 (24)
EPI – no. (%)		7 (44)	6 (40)	6 (50)	9 (53)
Body mass index (kg/m^2^)		22(20–28)	19 (18–23)	22 (19–24)	22 (20–25)

Percentages may not total 100 due to rounding. VAS: visual analogue scale. CP: Chronic pancreatitis. EPI: exocrine pancreatic insufficiency.

† Weak analgesics were defined as non-steroid anti-inflammatory drugs, paracetamole, codeine and tramadole. Strong analgesics were defined as opioid based therapies.

### Response to Pregabalin and Placebo Treatment

In the pregabalin group 16 of 31 patients (52%) were classified as responders compared to 12 of 29 patients (42%) in the placebo group (P = 0.45). A detailed analysis of the clinical endpoints was reported previously [11].

### Baseline QST Measurements

Baseline QST measurements are reported in Table 2 for the pregabalin group and Table 3 for the placebo group. The pregabalin responders group was hypersensitive to electric tetanic stimulation of the pancreatic area (ePDT ratio: 1.2 (0.9–1.3)) compared to non-responders group (ePDT ratio: 1.6 (1.5–2.0)) (*P* = 0.001). All other baseline QST measurements were comparable between responders and non-responders (Table 2 and Table 3).

**Table 2:** Baseline quantitative sensory testing measurements for the pregabalin treated group (n = 31).

		All patients (n = 31)	Responders (n = 16)	Non-responders (n = 15)	*P*-value*
pPDT (kPa)	Pancreatic area	155 (97–301)	155 (105–308)	146 (67–301)	0.37
	Control area	263 (142–329)	269 (213–329)	175 (120–329)	0.38
pPDT ratio	Pancreatic vs. control area	0.7 (0.5–1.0)	0.7 (0.5–1.0)	0.7 (0.6–1.0)	0.93
ePDT (mA)	Pancreatic area	5.1 (3.3–8.1)	4.7 (2.8–6.2)	6.8 (4.2–10.4)	0.06
	Control area	3.2 (2.3–5.1)	3.7 (2.7–5.1)	2.7 (2.2–5.6)	0.41
ePDT ratio	Pancreatic vs. control area	1.3 (1.0–1.8)	1.2 (0.9–1.3)	1.6 (1.5–2.0)	0.001†
CPM (%)		2 (−10–17)	4 (−17–19)	0 (0–4)	0.63
Cold pressor (sec)		38 (23–57)	27 (18–56)	43 (25–57)	0.24

* Responders vs. non-responders.

† Significant result after adjustment for multiple comparisons. pPDT, pressure pain detection threshold; ePDT, electric tetanic pain detection threshold; CPM, conditioned pain modulation.

**Table 3:** Baseline quantitative sensory testing measurements for the placebo treated group (n = 29).

		All patients (n = 29)	Responders (n = 17)	Non-responders (n = 12)	*P*-value*
pPDT (kPa)	Pancreatic area	159 (85–264)	243 (88–352)	143 (68–260)	0.29
	Control area	211 (106–380)	338 (125–458)	132 (97–341)	0.08
pPDT ratio	Pancreatic vs. control area	0.7 (0.5–0.9)	0.7 (0.5–0.8)	0.8 (0.4–1.3)	0.55
ePDT (mA)	Pancreatic area	5.4 (3.5–6.8)	5.6 (4.6–7.2)	3.8 (3.2–6.5)	0.35
	Control area	4.2 (2.4–5.9)	3.9 (2.9–5.0)	4.7 (2.0–6.2)	0.79
ePDT ratio	Pancreatic vs. control area	1.3 (1.0–1.5)	1.3 (1.0–1.7)	1.3 (1.0–1.3)	0.31
CPM (%)		12 (0–32)	16 (2–34)	10 (0–30)	0.85
Cold pressor (sec)		33 (23–98)	88 (25–180)	29 (20–46)	0.16

* Responders vs. non-responders. pPDT, pressure pain detection threshold; ePDT, electric tetanic pain detection threshold; CPM, conditioned pain modulation.

### Prediction of Pregabalin Effect

**Figure 2:**
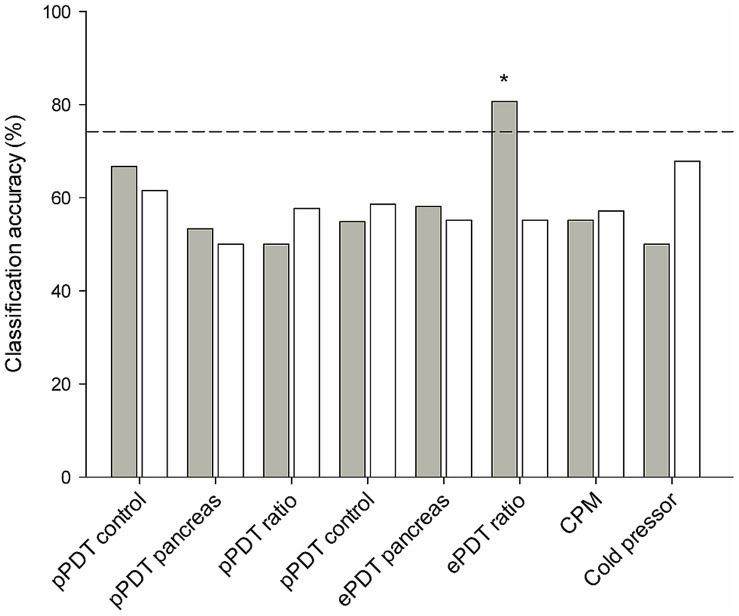
Predictive power of baseline QST measurements. The SVM was used to screen baseline QST measurements for their predictive power of analgesic response to pregabalin and placebo. Only the electrical pain detection tolerance (ePDT) ratio in the pregabalin treated group reached classification accuracy (80.6%) above chance level (74.2%; dotted line). CPM, conditioned pain modulation. *P = 0.02.

First, the different QST parameters in the pregabalin group were screened by the leave-one-out approach. The ePDT ratio was found to have the highest discriminatory power to separate responders from non-responders (Figure 2). The classification accuracy for this parameter was 80.6%, which was above chance level compared to random performance (*P* = 0.02). None of the other baseline QST measurements reached classification accuracy above chance level (Figure 2).

**Figure 3:**
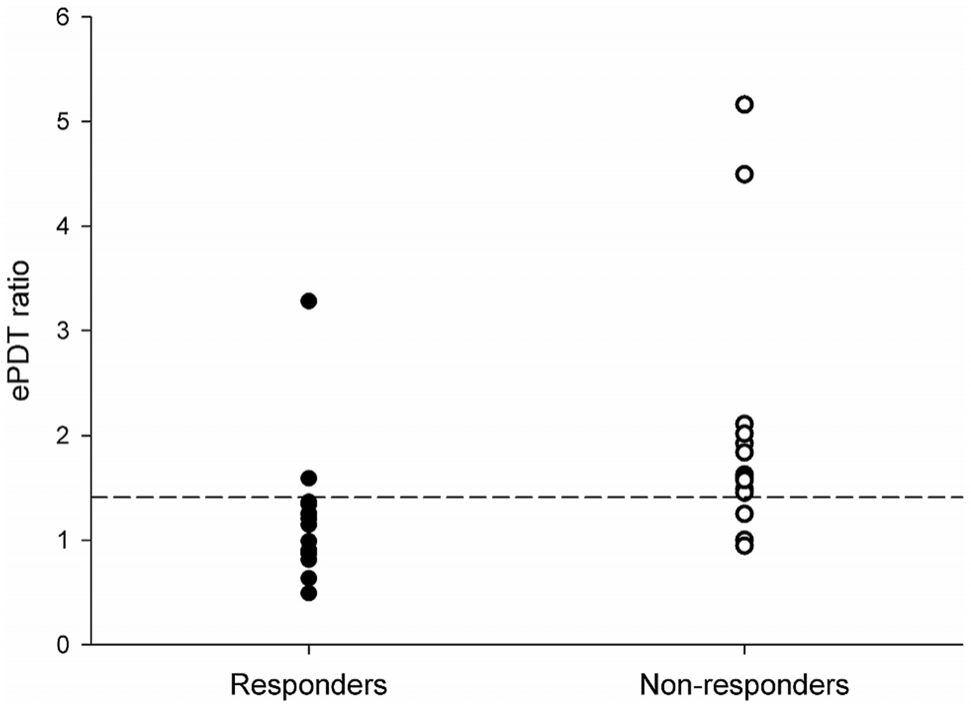
Electrical pain detection tolerance (ePDT) ratios for responders and non-responders in the pregabalin group. The optimal ratio to separate responder from non-responders was 1.41 (dotted line). This threshold separated groups with an accuracy of 83.9% (*P* = 0.007).

**Figure 4:**
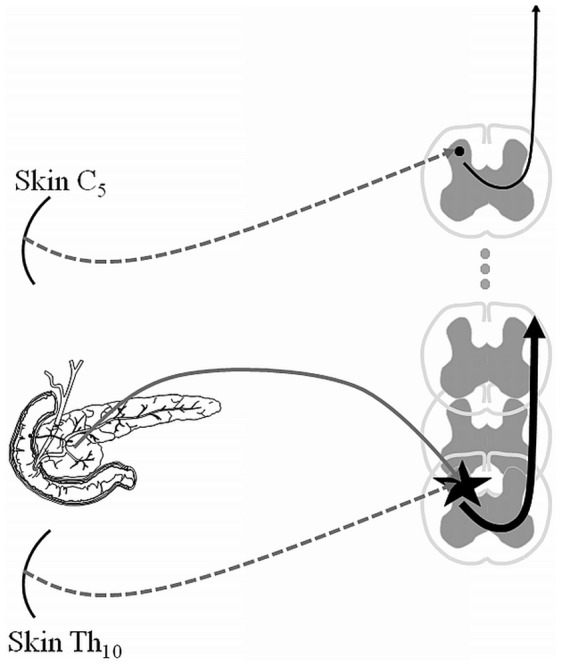
A simplified illustration of the experimental setup and findings. Increased afferent barrage from the pancreatic nerves results in central nervous system hyperexcitability (black star) predominantly at the lower thoracic segments. Visceral and somatic nerves converge on the same second order neurons and stimulation of the skin in this “viscerotome” (Th_10_) is amplified and interpreted by the brain as increased pain. Due to inter-individual differences in pain thresholds, normalization of Th_10_ thresholds was employed using a stimulus of the skin at C_5_ (control area) in the same subject. Pregabalin treated patients with high degree of central hyperexcitability, expressed as the electrical pain threshold ratio at Th_10_/C_5_ (ePDT ratio) <1.4, responded in 14 of 16 cases (87.5%) of cases, whereas patients with less hyperexcitability (higher ratio) responded in 3 of 15 cases (20%) (figure 3).

Next, data from all patients were used to train the SVM to determine the optimal ePDT ratio to separate responders from non-responders. The highest performance was found for an ePDT ratio of 1.41, with a corresponding classification accuracy of 83.9% (*P* = 0.007). For this threshold 14 of 16 patients were correctly classified in the responders group and 12 of 15 patients were correctly classified in the non-responders group (Figure 3). These numbers correspond to a sensitivity of 87.5% and a specificity of 80.0%. In figure 4 a simplified illustration of the experimental setup and findings is provided.

### Prediction of Placebo Effect

A similar approach as for the pregabalin group was used to screen QST parameters in the placebo group. The classification accuracy for the ePDT ratio was 55.2%, which was not above chance level compared to random performance (*P* = 0.80). In addition, none of the other baseline QST measurements in the placebo group reached classification accuracy above chance level (Figure 2).

## Discussion

We investigated the link between pre-treatment QST measurements and analgesic effect of pregabalin and placebo in patients with painful CP. Pregabalin effect was associated with pre-treatment sensitivity to electric tetanic QST. Hence, patients expressing lower pain thresholds in the pancreatic viscerotome compared to the control area are more likely to benefit from pregabalin treatment, whereas those with no difference in pain thresholds between the stimulation areas are less likely to benefit. These findings suggest sensitization of spinal neurons in the segment innervated by pancreatic visceral afferents to be an important predictor of pregabalin efficacy in patients with painful chronic pancreatitis. None of the QST parameters were associated with placebo analgesia.

### Methodological Considerations

Patients were instructed to report their daily average pain score in a pain diary, but no instruction was given concerning timing of daily pain score registration in relation to administration of study medication. This could potentially influence registration of clinical pain intensity. However, as pregabalin reach a steady state plasma concentration after maximal 48 hours, the analgesic effect would theoretically be constant throughout the day when administered two times daily [25]. This is supported by a recent publication where the pharmacokinetic profile of pregabalin was reported in patient with CP and found to be comparable to that of healthy subjects [26]. Also, patients were asked to report the average pain score for the last 24 hours and not the pain score at the time of registration. For these reasons we do not consider the timing of pain score collection to be of major importance.

The reliability of QST measurements has previously been reported in patients with CP [27]. Overall, sensory thresholds in the pancreatic viscerotome and control area are reproducible over time [27]. Conditioned pain modulation has been shown to be reproducible in test retest experiments in healthy volunteers [28], [29]. In contrast, a recent study demonstrated considerable variability of CPM in patients with CP [27]. Whether this phenomenon is due to impaired descending modulation in CP patients, as previously demonstrated in studies by our group, remains unknown and need further investigation [30], [31]. However, it may limit the usefulness of CPM for prediction of analgesic potency in CP patients.

An index (ePDT ratio) reflecting the relation between stimulation areas (i.e. pancreatic vs. control area) was derived to eliminate individual differences in pain thresholds. The ratio reflects the excitability of the neuronal pool with convergent projections from pancreatic visceral afferents, thereby providing an indirect measure of spinal neuronal excitability due to pancreatic nociceptive input [8]. Whether increased responsiveness in the pancreatic area reflected a true hyperalgesic state could not be determined from the present study since a healthy control group was not enrolled. However, several studies have documented hyperalgesia, and sensitization of second order neurons receiving convergent pancreatic input in patients with CP as compared to healthy controls [9], [30]–[32].

In order to obtain a clinical analogue to tonic pain and evoke maximal conditioning effect, a temperature of 2.0°C was used for the cold pressor paradigm [33]. Consequently, most subjects removed their hand due to intolerable pain intensity (i.e. VAS 10). For this reason, the cold pressor time was used for analysis instead of the VAS score to avoid a ceiling effect in the data.

The method for CPM assessment was chosen based on a previous review of the methodology of experimentally induced pain modulation, where the most sensitive models reported an average inhibitory effect of 40% [10]. In those studies the cold pressor test to an upper extremity was used as conditioning stimuli, in combination with mechanical stimulations of the lower body (test stimulus). Similar modalities were chosen for the present study, to obtain the widest possible dynamic range of CPM and to ensure maximum heterotopy between testing and conditioning stimulation.

### Prediction of Pregabalin Efficacy

The rationale for the present study was based on the hypothesis that analgesic agents used for CP should be prescribed by relating their mode of action to the specific patient’s pattern of pain processing. Thus, an agent targeting neuronal excitability, such as pregabalin, should be more efficacious in patients with evidence of neuronal sensitization. In agreement with this, we found that patients with segmental hyperalgesia of the pancreatic viscerotome, sharing spinal segmental innervation with the pancreas, had a superior clinical response to pregabalin treatment compared to patients with less pronounced hyperalgesia. This finding reflects the known mechanisms of action underlying pregabalin analgesia. Accordingly, *in vitro* studies indicate that pregabalin binds selectively to the alpha-2-delta subunit of voltage dependent calcium channels, thereby blocking the influx of calcium into presynaptic nerve terminals [15]. This in turn reduces release of excitatory neurotransmitters including glutamate, noradrenalin and substance P on spinal second order neurons, and thus dampens neuronal excitability [34]. In agreement with this, clinical studies have documented pregabalin’s analgesic efficacy in chronic pain disorders characterized by neuronal sensitization, including painful CP [11]–[14], [16]. In a recent trial we found this anti-nociceptive effect to be mediated primarily through sub-cortical mechanisms (i.e. spinal) [17]. This translates well to the findings of the present study where segmental hyperalgesia of the pancreatic viscerotome (reflecting spinal sensitization) was found to be associated with pregabalin efficacy.

Only electric stimulation was predictive of pregabalin efficacy, while pressure stimulation was not associated with clinical outcome. This finding is in line with a recent study from our group were pregabalin treatment resulted in a greater increase of electric pain thresholds than of pressure pain thresholds [31]. A possible explanation is that pregabalin is initially more effective in reducing skin sensitization, as reflected by electric thresholds, as compared to deep tissue sensitization, as reflected by pressure thresholds [35]. Pressure stimulations using the algometer potentially activate nociceptors in both the skin and in underlying muscle or bone. However, it has been shown that anesthetizing the skin will only result in minor changes in pressure thresholds and we consider the stimulus to predominantly reflect pain in deep structures [36].

Dynamic pain modulation (CPM) was not associated with pregabalin efficacy, which is an expected finding since pregabalin is not believed to target descending inhibitory pain modulation. Furthermore, as discussed above, CPM is not stable over time in CP patients, which may limit its usefulness for prediction of analgesic potency in CP patients [27], [30].

### Clinical Implications

The experimental protocol presented in the current study provides a non-invasive technique to identify patients with a specific pattern of abnormalities in central pain processing. This approach can be used to support clinicians when establishing treatment indications in patients with pancreatic pain (or other chronic pain disorders). Most analgesics are only effective in a subset of patients and many have adverse effects [37]. The mechanism-based approach presented here may thus help to prevent a long and often painful trial and error process of finding an appropriate therapy for the individual patient. Of particular interest, preoperative evaluation of central pain processing could also be a useful biomarker to identify those CP patients who will not benefit from endoscopic or surgical interventions. Hence, patients with evidence of severe central sensitization may have a lower chance of successful outcome to surgery or endotherapy compared to patients with less sensitization [19].

### Conclusions

The present study provides first evidence that QST predicts the analgesic efficacy of pregabalin in patients with painful CP. The method thus carries the potential for shortening a long and painful trial and error process of finding an appropriate therapy for the individual patient and thus comprises a significant step towards personalized pain medicine.
